# Molnupiravir: A Versatile Prodrug against SARS-CoV-2 Variants

**DOI:** 10.3390/metabo13020309

**Published:** 2023-02-20

**Authors:** Divya Teli, Pankti Balar, Kishan Patel, Anu Sharma, Vivek Chavda, Lalit Vora

**Affiliations:** 1 Department of Pharmaceutical Chemistry, L. M. College of Pharmacy, Ahmedabad 380009, India; 2 Pharmacy Department, L. M. College of Pharmacy, Ahmedabad 380009, India; 3 Department of Chemistry, University at Buffalo, Buffalo, NY 14260, USA; 4 Department Pharmaceutical Sciences, University of Massachusetts, Boston, MA 02125, USA; 5 Department of Pharmaceutics and Pharmaceutical Technology, L. M. College of Pharmacy, Ahmedabad 380008, India; 6 School of Pharmacy, Queen’s University Belfast, 97 Lisburn Road, Belfast BT9 7BL, UK

**Keywords:** prodrug, molnupiravir, *N*4-hydroxycytidine, omicron, patients with comorbidity

## Abstract

The nucleoside analog β-D-*N*4-hydroxycytidine is the active metabolite of the prodrug molnupiravir and is accepted as an efficient drug against COVID-19. Molnupiravir targets the RNA-dependent RNA polymerase (RdRp) enzyme, which is responsible for replicating the viral genome during the replication process of certain types of viruses. It works by disrupting the normal function of the RdRp enzyme, causing it to make mistakes during the replication of the viral genome. These mistakes can prevent the viral RNA from being transcribed, converted into a complementary DNA template, translated, or converted into a functional protein. By disrupting these crucial steps in the viral replication process, molnupiravir can effectively inhibit the replication of the virus and reduce its ability to cause disease. This review article sheds light on the impact of molnupiravir and its metabolite on SARS-CoV-2 variants of concern, such as delta, omicron, and hybrid/recombinant variants. The detailed mechanism and molecular interactions using molecular docking and dynamics have also been covered. The safety and tolerability of molnupiravir in patients with comorbidities have also been emphasized.

## 1. Introduction

The pandemic caused by the coronavirus disease (COVID-19) has had a substantial impact on human populations, resulting in significant morbidity and mortality [1,2,3,4,5,6,7,8]. Globally, there are 636 million reported cases of COVID-19 (as per WHO) [9]. Severe acute respiratory syndrome coronavirus 2 (SARS-CoV-2) changes structurally over time due to mutation. These changes affect the spread ability and susceptibility of a virus to infection, disease severity, performance of therapeutic agents, diagnostic methods, and ultimately public health [10,11,12]. Since the outbreak of COVID-19, approximately 4000 different genetic changes have been detected in the virus responsible for the disease, known as SARS-CoV-2 [13,14]. Variants of concern (VOCs) are types of SARS-CoV-2 that have undergone changes in the proteins on their surface, specifically the spike glycoprotein and the receptor binding domain (RBD) to human angiotensin converting enzyme 2 (*h*ACE2); consequently, the binding affinity of RBD with *h*ACE2 will be increased, and thus, viral transmission and virulence will also be increased. These changes can affect the virus’s behavior and its potential to cause illness [15,16,17]. VOCs include Alpha, Beta, Gamma, Delta, and Omicron subvariants [18,19,20]. As the SARS-CoV-2 mutation rate is high, therapy becomes challenging. Some antiviral agents, such as remdesivir, paxlovid (nirmatrelvir/ritonavir), and molnupiravir, are authorized for the management of COVID-19 [21,22]. The WHO also recommends the emergency use of COVID-19 vaccines such as Covishield (Serum Institute of India Pvt. Ltd., Pune, India), Comirnaty (BioNTech Manufacturing GmbH, Berlin, Germany), Vaxzevria (AstraZeneca AB, Cambridge, UK), Spikevax (Moderna Biotech, Cambridge, MA, USA), CoronaVac, Covaxin, Nuvaxovid (Novavax CZ, Jevany, Czechia), and Convidecia (CanSino Biologics Inc., Tianjin, China) [23,24,25]. Despite significant progress in COVID-19 therapeutics, precise treatment has yet to be discovered.

A prodrug is defined as a biologically inactive compound that, after administration in the body upon biotransformation, is converted into a biologically active entity. Prodrugs can be considered a well-established domain of medicinal chemistry that serves as a vital platform for rational drug design and discovery [26,27]. The primary reasons for the market demand for prodrugs are to achieve targetability [28], desired solubility [29,30], prolonged duration of action [31], and reduced toxicity [32]. Prodrugs are necessary for anti-SARS-CoV-2 therapeutics, which play a major role in combating COVID-19 [33]. Antiviral prodrugs have been tolerated in different aged COVID-19 populations, showing susceptibility toward different variants, including delta and omicron [34,35].

There are several types of antiviral prodrugs based on their mechanism of action, like enzyme-activated prodrugs and pH-activated prodrugs. Enzyme-activated prodrugs are activated by enzymes present in the body, such as fructose-1,6-bisphosphate aldolase in the case of favipiravir and carboxylesterase in the case of molnupiravir. These prodrugs are designed to be selectively activated in the body, allowing for targeted delivery of the active drug. On the other hand, pH-activated prodrugs are activated by changes in the pH of the body. An example of this type of prodrug is remdesivir, which is activated in acidic environments, such as lysosomes. Remdesivir was approved first for the treatment of COVID-19 on 1 May 2020 [36]. Initially, conducted clinical trials (NCT04280705, NCT04292730) showed the efficacy of remdesivir for the treatment of COVID-19 [37,38]. In addition, according to trial NCT04257656, it was speculated that remdesivir was not associated with statistically significant clinical benefits in adult, hospitalized severe COVID-19 patients [39]. Remdesivir was parenterally administered to the patients, and it was very expensive. Apart from this, favipiravir, another nucleoside prodrug, was not approved by the US FDA; however, it is approved in Russia and China. A high dose is required to exert antiviral action. Paxlovid, a peptidomimetic drug, encountered significant drug–drug interactions as ritonavir is a potent CYP3A inhibitor [40]. Another orally available nucleoside prodrug, molnupiravir, was found to exert potent anti-SARS CoV-2 activity in *in vitro* and *in vivo* trials [41], although it has safety concerns. Additionally, it has also demonstrated activity against the omicron variant of SARS-CoV-2, which is explained in Section 5 of this article. Table 1 compares the antiviral therapeutics of COVID-19.

Molnupiravir is an oral ribonucleoside analog, which means it is similar in structure to one of the building blocks of RNA, a molecule that is important for the replication of viruses such as SARS-CoV-2 [52]. *N*4-hydroxycytidine (NHC, EIDD 1921) is a new isobutyryl ester prodrug that has been tested in phase II and III studies to treat SARS-CoV-2 infection [53]. The US FDA granted emergency use authorization of molnupiravir for the treatment of mild moderate to severe COVID-19. Molnupiravir targets RNA-dependent RNA polymerase (RdRp) and hinders the RNA-dependent translation and transcription of viral RNA by introducing copying errors during the process of replication [54]. Molnupiravir, a potential antiviral drug, has been shown to inhibit the spread of SARS-CoV-2 in animal models [55]. It has a significantly higher ability to neutralize the virus than other antiviral prodrugs [56]. These findings suggest that molnupiravir may be a promising option for treating infections caused by SARS-CoV-2.

Molnupiravir stands out as an exceptional prodrug for treating COVID-19 due to its numerous benefits. Unlike other antiviral drugs that are administered through injections or intravenous infusion, molnupiravir can be taken orally, which makes it more convenient for patients. It is rapidly converted into its active form after oral administration, which leads to a rapid onset of action and improved efficacy. It is selectively activated by carboxylesterases and has high potency against SARS-CoV-2. In this review article, the primary focus is on molnupiravir, a nucleoside prodrug, and its active metabolites, which exhibit promise as broad-spectrum anti-SARS-CoV-2 agents due to their efficacy against various SARS-CoV-2 strains, including the delta and omicron variants. The mechanism of action for molnupiravir triphosphate (MTP) has been explained, with particular attention given to the key interactions of MTP with the viral RdRp through a molecular docking approach. Additionally, the article critically evaluates the safety and tolerability of molnupiravir in patients with comorbidities.

## 2. Mechanism of Action of Molnupiravir

**Figure 1:**
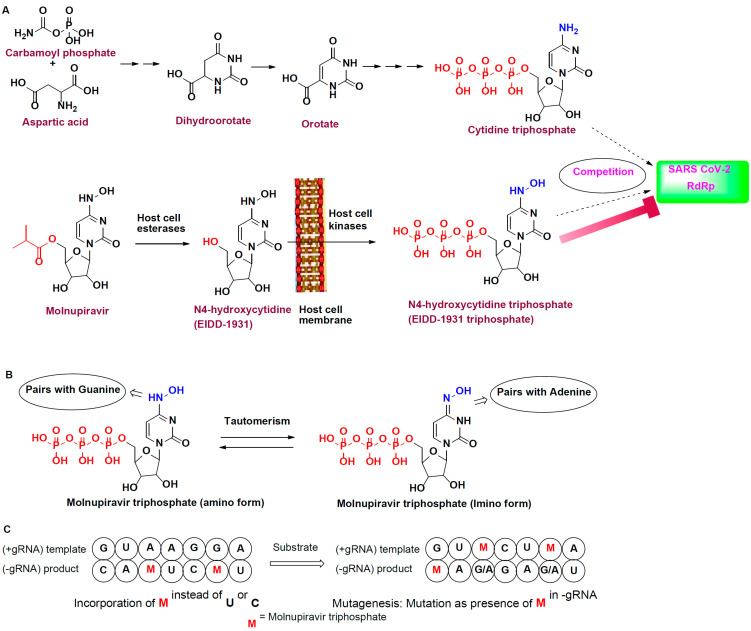
Mechanism of action of molnupiravir. (**A**) Competitive inhibition of RdRp by MTP. (**B**) Tautomer of MTP coupled with guanine and adenine. (**C**) Mutation in the presence of MTP in progeny RNA.

As discussed, molnupiravir is a prodrug. It is first hydrolyzed into *N*4-hydroxy cytidine (NHC) by host cell esterase [57,58]. The resulting alcohol analog is phosphorylated to obtain the active *N*4-hydroxycytidine triphosphate (EIDD-1931 triphosphate) [59]. This phosphorylated analog structurally resembles the endogenous substrate cytidine or uridine triphosphate for RdRp (Figure 1A). Thus, EIDD-1931 triphosphate competitively inhibits RdRp and further inhibits viral replication [60,61].

Complementary base pairs suggest that G:C and A:U or A:T pairs are found in the structure of nucleic acids [62]. MTP can attain two tautomeric forms: amino and imino forms. Because of its structural resemblance to the nitrogenous base of nucleic acids, the amino form of MTP has the capability to pair with G (M:G), and the imino form can pair with A (M:A) by forming hydrogen bonds (Figure 1B) [63]. Thus, MTP is used as a template for protein biosynthesis (Figure 1C) [64,65]. The goal is to prevent the production of viral proteins, which would result in the death of the viral cell [66].

## 3. Chemistry of Molnupiravir

Molnupiravir is a ribonucleoside prodrug composed of two structural fragments—*N*-hydroxycytosine and a ribose sugar. It has a molecular weight of 329.31 g/mol and a molecular formula of C_13_H_19_N_3_O_7_.

**Figure 2:**
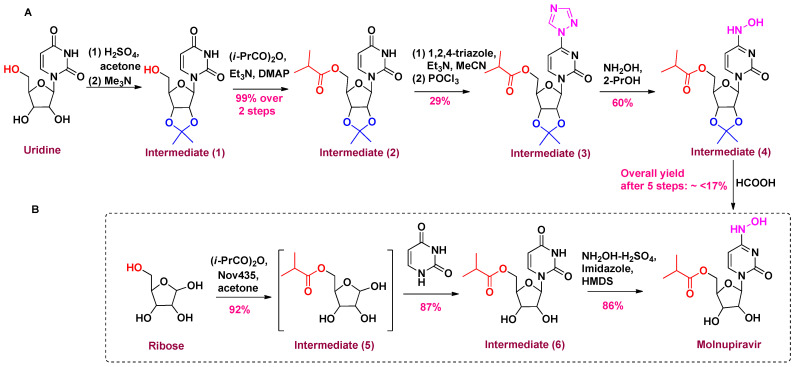
Synthetic schemes for the synthesis of molnupiravir. (**A**) Original synthetic route from uridine reported by Emory University. (**B**) Potential synthetic route from ribose reported by Merck.

Various synthetic procedures utilize uridine, cytidine, or ribose as key starting materials. Emory University reported the first synthetic approach, which consisted of five stages starting with uridine as a precursor, showed a very poor yield, and used the expensive reagent 1,2,4-triazole (Figure 2A). The vicinal diol was secured by acetone and H_2_SO_4_ to afford acetonide (1) in the first step. It was then treated with isobutyric acid anhydride in the presence of 4-dimethylaminopyridine (DMAP) and triethylamine to generate the isobutyryl ester (2). Phosphorous oxychloride (POCl_3_) was added for chlorination of the hydroxyl group followed by the addition of a high equivalent of 1,2,4-triazole to form the triazole analog (3). This conversion had a very low yield (29%). The triazole (3) was then substituted using hydroxylamine analog (4) followed by deprotection of acetonide using formic acid to afford molnupiravir. The overall yield after five steps was very poor [67].

By using continuous flow chemistry and reordering reaction sequences, the synthesis yield was increased up to 61% [68]. Two common impurities were also observed in this route: One is raised from the hydrolysis of isobutyrate ester; and the other is formed by replacement of hydroxylamino with hydroxyl while deprotecting acetonide under strongly acidic conditions. Biocatalytic synthesis routes have also been reported [69,70,71]. All the reported routes showed significant improvements compared to the original route. A potential, facile, high-yield, and low-cost commercial manufacturing route was developed by Merck and Codexis [72]. It uses a three-step process containing ribose as a key starting material (Figure 2B). Ribose was first esterified enzymatically. The next step required the usage of four enzymes—phosphorylation by MTR kinase, nucleobase formation by uridine phosphorylase, revitalization of acetyl phosphate and ATP by acetate kinase, and pyruvate oxidase to yield the penultimate intermediate (6). These enzymes were used at low burdening ranging from 0.2 to 9 wt %. The acidic carbonyl of (6) was then converted into hydroxylamine to afford molnupiravir in the last step. The Merck group seems promising to fulfil the need worldwide.

## 4. Molecular Docking Study

We studied the molecular interaction of the active form of molnupiravir with RdRp using a molecular docking method. It was performed using Schrödinger Maestro 11.8. The 3D crystal structure of RNA-dependent RNA polymerase (RdRp) with a PDB code of 7BV2 [73] was downloaded from the Protein Data Bank (https://www.rcsb.org/ (accessed on 30 September 2022)). It was prepared using the protein preparation wizard tool. The grid was generated by taking the centroid of the cocrystal ligand (remdesivir triphosphate) using a receptor grid generation panel. The structure of MTP was prepared using the LigPrep tool. The extra precision (XP) algorithm of the Glide tool was used to study the key interactions.

**Figure 3:**
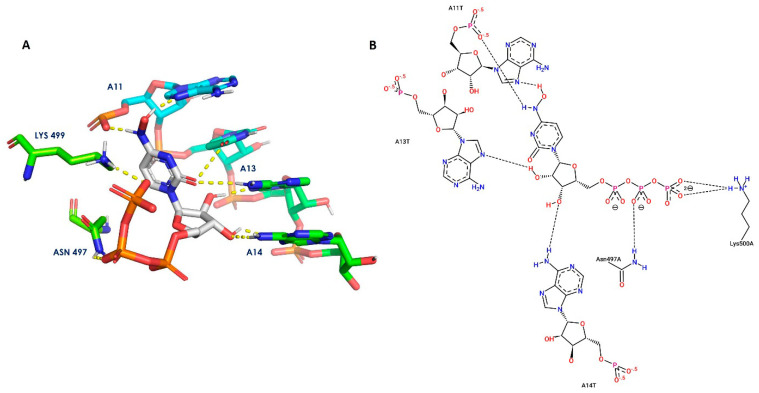
Receptor–ligand interaction diagrams. (**A**): 3-D interaction of MTP with the active sites of RdRp; MTP is shown as gray sticks, and receptor amino acid residues are shown as atom-type colored sticks. Yellow dotted lines highlight the hydrogen bonds that MTP and the receptor constructed. In panel (**B**), MTP interacts in two dimensions with the active sites of RdRp.

Key interactions were visualized using PyMoL and PoseViewer (Figure 3). A docking score of −9.67 kcal/mol was observed. MTP interacts with RdRp by forming hydrogen bonds with Asn497 and Lys499. MTP also interacts with RNA residues, adenosine triphosphate A11, A13, and A14. This docking result suggests that MTP has high binding potential to RdRp. Thus, this *in silico* study provides a basis for the potential significance of administering MTP to patients infected with COVID-19.

Analogously, many researchers have investigated the similar interactions of molnupiravir with RdRp using a molecular docking approach in various computational softwares. One such recent study, reported by Sharov et al., explained the docking study of three plausible tautomers of molnupiravir with RdRp using AutoDock Vina 1.2.0 [74]. The keto oxime, hydroxylamine, and keto-hydroxylamine structures exhibited −9.9, −9.3, and −7.9 kcal/mol docking scores, respectively. DFT calculations were also carried out to verify the structures of these tautomers, and the keto-oxime tautomer was found to be the most energetically stable.

Additionally, Celik et al. performed homology modeling to construct the RdRp of Delta subvariant AY.4 (having the mutation at the NSP12 site) using Chimera 1.16 and evaluated the molecular interaction of molnupiravir against delta subvariant AY.4 using Schrödinger Maestro 12.8 [75]. The molecular docking study revealed that MTP binds more predominantly to the RdRp of subvariant AY.4 (XP glide docking score: −10.28 kcal/mol) than to the RdRp of wild-type SARS-CoV-2 (XP glide docking score: −8.39 kcal/mol). This finding was also supported by molecular dynamic simulations carried out over 100 ns using Gromacs. Thus, this *in silico* study hypothesized that MTP is also beneficial to patients carrying the Delta subvariant AY.4 [75].

## 5. Impact of Molnupiravir on SARS-CoV-2 Variants

The SARS-CoV-2 pandemic remained uncontrolled in the initial phase of its development due to its strong binding affinity, contemporary variants, and rapid mutation rate. Recently, molnupiravir was preclinically and clinically evaluated for its efficacy and potency against delta and omicron variants.

### 5.1. Preclinical Studies of Molnupiravir

Vangeel et al. conducted an *in vitro* cell assay to evaluate the antiviral activity of molnupiravir and its metabolite EIDD-1931 against the ancestral SARS-CoV-2 and five other CoVs, including the delta and omicron variants, using VeroE6-GFP cells. The EC_50_ results showed that these molecules were equally effective against both delta and omicron variants, with a maximal change in median EC_50_ of less than 2.5 times [76]. Wahl et al. conducted a study to examine the effects of EIDD-2801 on immunodeficient mice implanted with human lung tissue. The drug was administered at 24 h or 48 h after SARS-CoV-2 exposure, and then it was administered every 12 h. The study concluded that molnupiravir was therapeutically and prophylactically beneficial [77].

In a study by Lieber et al., it was found that the effectiveness of molnupiravir varies according to VOC type and biological sex using human airway epithelial organelles, mice, and a dwarf hamster model of severe COVID-19-type lung injury [78]. In a study on omicron infection in dwarf hamsters, it was found that the infection was more common in male hamsters than in female hamsters, suggesting that there may be a significant difference in the susceptibility to the infection between the two sexes. This difference may be due to the differences in production of certain immune factors or other biological factors that affect the ability to fight off the infection. The study also found that the severity of the infection in hamsters was influenced by the presence of certain viral proteins, known as virulence factors. Although pathogenicity in hamsters depended on VOCs, a noticeable reduction in viral load at a ratio of 1:4 was observed for delta and gamma variants after treatment with molnupiravir. Treatment of ferrets with molnupiravir effectively prevented the transmission of COVID-19 infection. The outcome indicates that molnupiravir affects all the sub variants [78].

Similarly, Rosenke et al. also conducted a study to evaluate the effectiveness of molnupiravir, both as a standalone treatment and in combination with nirmatrelvir, for treating mild to moderate infections in rhesus macaques. The study involved administering molnupiravir and nirmatrelvir to infected rhesus macaques and measuring the effectiveness of the treatments in reducing the severity of the infections. The results of the study were used to determine the potential usefulness of molnupiravir and molnupiravir/nirmatrelvir as treatments for infections in humans [79]. The combination of molnupiravir and other drugs has been shown to be effective in reducing the severity of the disease and decreasing the amount of virus present in the respiratory system. In a study conducted by Jeong et al., the combination of molnupiravir and remdesivir led to a moderate improvement in survival rates of infected mice, while the combination of molnupiravir and nirmatrelvir resulted in a significant improvement in survival rates up to 80%. These findings suggest that the combination of molnupiravir with certain other drugs may have a synergistic effect, meaning that the combination is more effective at reducing the severity of the disease than either drug alone [80].

### 5.2. Clinical Studies of Molnupiravir

Molnupiravir in clinical trials was previously reviewed by Singh et al. [81]. One of the double-blind phase 1 clinical studies (NCT04392219) was proven to be safe and well tolerated with a daily tolerable dose of up to 1600 mg without any serious major side effects for up to 5.5 days in healthy individuals. Compared to placebo, patients treated with a dose of 800 mg twice daily of molnupiravir in a phase 2 clinical study (NCT04405584) showed significantly reduced recovery time.

Phase 1 clinical trial data for EIDD-1931 (NCT04392219) showed that the compound has a half-life of approximately 60 min and a delayed clearance phase after large single doses or repeated doses. Multiple doses of EIDD-1931 did not result in accumulation of the drug in the body [82]. The most commonly reported adverse events in studies using single ascending doses and multiple ascending doses are headache and diarrhea, respectively. It is worth noting that patients receiving placebo experienced more side effects compared to those receiving molnupiravir. In this instance, the excretion of low levels of EIDD-1931 in urine is likely due to its metabolic conversion to cytidine and uridine, which differs from other analogs and natural nucleosides that typically undergo elimination through the kidneys [82].

A phase 2a study found that the most effective dose of molnupiravir for reducing the amount of virus in the body is 800 mg. Hospitalization decreased by 7.2% in patients administered molnupiravir compared to placebo [41]. The risk of death also decreases by 89% compared to placebo. Another case study was performed under the name “MOVe-IN (NCT04575584)”, and the results are summarized here [83]. Three molnupiravir dosage levels given twice daily for five days to patients hospitalized for COVID-19 did not result in dose-limiting side effects similar to placebo. There were no safety issues with molnupiravir during the five and fourteen days, including any adverse events that resulted in death. Additionally, there was no proof of hematologic, pancreatic, or hepatic toxicity during this acute-care trial’s safety assessment period. The majority of the deaths were observed in people who were older, had underlying comorbidities, and/or had significant COVID-19 at baseline, and the majority looked to be related to COVID-19 problems.

Despite the fact that each molnupiravir group had a larger percentage of patients who experienced fatal adverse events compared to the placebo group, the overall number of fatalities was low. With equal median recovery durations for both the active and placebo groups, sustained recovery rates—the primary end point—were not observed in the hospitalized subjects who were the subject of the study. Published data showing the clinical and virologic effectiveness of postoperative plasma and monoclonal antibodies often used in the patient setting during early disease (average time since symptom onset: 2 to 4 days) further support the necessity of early antiviral treatment in COVID-19, while the same therapeutic interventions, with certain anomalies, certainly lack those perks when used in hospital admissions. A phase 3 clinical study evaluated the safety measure along with the therapeutic efficacy. They suggested that 800 mg of drug is safe enough to be consumed twice a day. It was concluded that molnupiravir decreases the clinical symptoms and rapid recovery from COVID-19 infection (proved by negative RT–PCR) [84].

A recent study of hospitalized patients receiving drugs such as molnupiravir or nirmatrelvir-ritonavir combination therapy was reported by Wong et al. in August 2022. The data for the study were collected from different sources, such as hospital authorities and national sources. The participants for the study were divided into four different groups, namely, patients receiving molnupiravir, nirmatrelvir-ritonavir combination therapy and their matched controls. A lower mortality rate, lower risk of disease progression and redundancy in oxygen therapy were reported in the antiviral treatment groups compared to the matched controls. However, no significant difference in the need for IMV or intensive care was observed in severely infected patients with SARS-CoV-2 [85]. Zou et al. demonstrated the safety and efficacy of molnupiravir against the omicron variant through a randomized clinical study. It was observed that the time of clearance was reduced by molnupiravir compared to the control [86].

Thus, various clinical studies conclude that treatment of patients with molnupiravir can reduce the risk of COVID-19 infection and increase the survival rate of those who are moderately infected by SARS-CoV-2. However, it is deemed to be ineffective in later stages of severe COVID-19 infection.

A study by Tiseo et al. suggested that the occurrence of death corresponding to COVID-19 was higher with remdesivir than with molnupiravir or nirmatrelvir/ritonavir [87]. Molnupiravir was found to be well tolerated when COVID-19 treatment was carried out on older patients and those with vaccination and comorbidities [88]. It was also reported by Wong et al. that oral antiviral therapy containing molnupiravir or nirmatrelvir/ritonavir was proven beneficial for the treatment of COVID-19 caused by the SARS-CoV-2 omicron (B.1.1.529) variant, which also reduces the risk of mortality and hospitalization [89]. The impact of molnupiravir on SARS-CoV-2 variants of concern have been summarized in Table 2. 

## 6. Pharmacovigilance Profile of Molnupiravir

### 6.1. Adverse Effects of Molnupiravir

According to National Health Service (NHS) statistics, these typical molnupiravir adverse effects affect more than 1 in 100 people: vertigo, headache, and diarrhea. *In vitro* investigations show that neither molnupiravir nor its active metabolite NHC inhibit or promote key drug-metabolizing enzymes or major drug transporters [98]. To date, molnupiravir has not been found to have any drug–drug interactions. It should be studied further.

The use of molnupiravir in unvaccinated, out-of-hospitalized persons with COVID-19 who were recruited soon after symptom onset (5 days) was investigated in the international MOVe-OUT Phase 3 study (NCT04575597). The experiment took place before the Omicron variant and its subvariants were discovered. Men made up 49% of the participants, white people made up 57%, Hispanic people made up 50%, and black people made up 5%. Three days passed between the start of COVID-19 symptoms and randomization for 48% of the subjects [99].

Molnupiravir treatment minimized the risk of rehabilitation or death by 31% by day 29. There were one and nine fatalities in the molnupiravir and placebo arms, respectively. No major adverse events were observed in either odd group. A subsequent examination of data from trial patients found that using molnupiravir reduced the probability of needing respiratory interventions by 21% [100].

Early therapy lowers the risk of hospitalization or mortality for COVID-19 individuals who are exposed to the prodrug molnupiravir, which has antiviral action. According to Nakamura et al., this was usually well-tolerated after being administered in single and repeated doses [101]. The drug was permitted to be utilized in Japan under the “special approval for urgency” system based on trial outcomes, as well as universal phase II and phase III data. There were no reports of serious adverse effects, except that only three participants experienced skin eruption. One participant in research of non-Japanese people experienced similar discomfort in the form of rash. These negative side effects fluctuated in severity from mild to moderate and were medicated with steroids and antihistamines to recover.

To support the approval of a new medication for use in Japan, it was deemed necessary to gather additional safety data from healthy Japanese volunteers. The current phase I trial included a total of 20 patients who received a second dose of the medication (molnupiravir) and 5 patients who received a placebo. Of the 1433 participants in the worldwide phase III study, only 8 were Japanese. The phase I trial, which involved administering the medication at a therapeutic dosage of 800 mg every 12 h, provided regulatory agencies with additional data on the safety and durability of the medication in treating COVID-19 [102].

Molnupiravir is easier to administer in an outpatient setting, requires only brief oral administration, and thus has higher compliance. The benefit of a medicine such as molnupiravir is that, in contrast to other EUA authorized treatments for COVID-19, made on a higher scale, it does not demand cold storage and may be administered outside of hospitals. This implies that the medication must also be highly cost-effective and safe to be used widely. Potentially, from a safety perspective, even while its mutagenesis capability is a blessing for decreasing viral RNA, this could turn into a curse by creating additional mutations in the virus that lead to increased resistance. Safety concerns still persist since study participants were mandated to keep abstinence or take contraception due to worries about a congenital defect if they became pregnant. However, the short usage of 5 days makes this seem quite implausible [103]. The drug interaction potential of molnupiravir is summarized in Table 1.

### 6.2. Cases of Molnupiravir in Patients with Comorbidity

To study the efficacy and safety of molnupiravir in patients with comorbidities, a preliminary clinical study was performed and evaluated in kidney transplant recipients by Villamarín et al. [104]. Nine kidney transplant recipients with SARS-CoV-2 (omicron variant) infection were subjected to molnupiravir and were compared with remdesivir treatment with similar patients. The molnupiravir-treated patients had a good clinical evaluation with a lower risk of hospitalization and no evidence of serious adverse reactions. None of the remdesivir-treated patients progressed in COVID-19 severity. Molnupiravir was well-tolerated at the renal level and found no interactions with immunosuppressants during therapy. According to one of the real-world data reported by Czarnecka et al., a total of 107 patients (including comorbid conditions) were enrolled for the treatment of COVID-19 with molnupiravir. Approximately half (43.9%) of the participants had two risk factors, 26.1% had three risk factors, and 18.7% had four risk factors for COVID-19. Chronic kidney disorders and immunosuppressive therapy can be identified as the most predominant risk factors for COVID-19 severity. Herein, 49 patients were suffering from various stages of chronic kidney disorders, and 72 patients were receiving immunosuppressive treatments. The study stated that molnupiravir was safe and that it constitutes an alternative for such risk factors for COVID-19 [105]. Additionally, Suzuki et al. reported a study utilizing 1929 micron-infected COVID-19 patients’ real-world data [106]. After 1:3 propensity score matching, clinical deterioration was compared between molnupiravir users (230 participants) and nonusers (690 participants). The high-risk factors comprise dyslipidemia and hypertension. It was stated that molnupiravir treatment should be considered to prevent deterioration in high-risk patients with mild-to-moderate COVID-19.

Two more clinical trials have been identified by the Merck group for evaluating the efficacy of molnupiravir in renal impairment (NCT05386758) and hepatic impairment (NCT05386589). These studies are currently in recruitment.

### 6.3. Study of Molnupiravir in Vaccinated and Unvaccinated Patients

AGILE CST-2 (NCT04746183) was a phase 2 clinical trial that evaluated the safety and effectiveness of molnupiravir in individuals who had received COVID-19 vaccinations [107]. The study included 180 participants, with an average age of 43 years and approximately equal numbers of males and females. Of the participants, 90 had received at least one dose of a COVID-19 vaccine 14 days prior to enrolling in the trial. The study found that both the molnupiravir and placebo groups had similar rates of adverse events, with 200 and 219 events reported in the molnupiravir and placebo groups, respectively. Four of the 90 participants in the placebo group experienced serious adverse events, all of which required hospitalization. There was also a total of four hospitalizations in the placebo group, with three occurring on days 15 and 29 and one requiring oxygen for two days. In contrast, 73 of the 90 molnupiravir participants had at least one adverse event by day 29. This trial suggests that molnupiravir may be safe for use in individuals who have received COVID-19 vaccinations, although further research is needed to fully understand its potential therapeutic effects [107].

In another study conducted on the effect of molnupiravir on omicron variants, 90% of the study population was vaccinated with a mostly inactivated vaccine. They studied the antibody-titter-neutralizing property as a core element to differentiate and found that there was no significant difference in patients with or without immunization. It was observed that even T-cell-mediated immunity remained unaltered. This study shows the superiority of molnupiravir over the immunity attained by vaccination [108]. H Khoo et al. suggested that when compared to baseline, a decrement of 4.8 log_10_ copies was observed on administration of molnupiravir. It was observed that the log_10_ copy decrement was 5.4 and 4.1 for vaccinated and placebo control, respectively, which was 1.2 log_10_ times higher. This suggests that the effect of vaccination synergizes with the antiviral property of molnupiravir [109].

In February 2022, a phase III, double-blind, randomized, placebo-controlled trial (NCT04575597, MOVe-OUT) was reported by Bernal et al. in 1433 mildly to moderately infected, unvaccinated adult SARS-CoV-2-infected patients to evaluate molnupiravir treatment. Two different groups received 800 mg of molnupiravir or placebo twice daily for 5 days. At the end of day 29, patients treated with molnupiravir had a significantly reduced risk of hospitalization (7.3%) compared to placebo (1.1%). Adverse events were more prominent in placebo-treated patients than in drug-treated patients. At the end of day 29, 1 death was disclosed in patients treated with molnupiravir and 9 deaths in patients treated with placebo [110].

### 6.4. Pharmacokinetic Studies of Molnupiravir

Pharmacokinetic studies play a crucial role in achieving the ultimate goal of developing a clinical candidate [111]. To date, several studies have been published on the pharmacokinetic parameters (absorption, distribution, metabolism, excretion, and toxicity) of molnupiravir in animals and humans. A few of them have been discussed here. Painter et al. conducted a placebo-controlled double-blind ascending dose-randomized trial on 64 COVID-19 patients and 54 healthy persons and evaluated the pharmacokinetic parameters of molnupiravir and found that it was well-absorbed between doses of 50 and 1600 mg when administered orally. The NHC was found to have better oral bioavailability compared to its prodrug, molnupiravir. Although absorption was slower in the fed state, with lower t_max_ and C_max_ values, the extent of absorption was comparable in both fed and fasted states [112]. Molnupiravir has no detectable protein binding. *In vivo* kinetic studies performed on mice indicate the dose-dependent exposure of the active form of molnupiravir in the respiratory tissues [113]. Similarly, when the drug was administered orally in ferrets, the active form, MTP, was found to be sustained (>3.2 nmol/g of lung tissues) in the respiratory tissues for over 8 h of administration [114]. During the *in vitro* metabolic studies reported by the European Medicines Agency, molnupiravir was found to be relatively unstable in mouse, rat, and monkey plasma (t_½_: 0.4 h) but more stable in dog and human plasma (t_½_: 3.2 h and 1.05 h, respectively) [115]. The *in vivo* metabolism of the drug was investigated in rats and dogs by orally administering radiolabeled molnupiravir solution in 1% methylcellulose, adjusting the dose to 30 mg/kg. The majority of the dose is well absorbed in the body (approximately 90%) and eventually metabolizes NHC, MTP, and then pyrimidine metabolites. These metabolites can be detected using modern hyphenated analytical techniques. Amara et al. developed such an LC–MS/MS method and validated it for the simultaneous quantification of molnupiravir and NHC in biological fluids [116].

Molnupiravir is accompanied by caution about potential embryo-fetal toxicity [117]. It is advised by medical practitioners that individuals with child-bearing potential, both male and female, should use reliable contraceptives during treatment and continue the same for approximately 3 months after treatment. Molnupiravir is not advised during pregnancy. As per the available preclinical data, a high dose (eight-fold higher than the standard) administered in the pregnant rat model was found to have teratogenicity and developmental toxicity. When an 18-fold higher dose was administered to a pregnant rabbit model, fetal growth was found to be reduced. Limited data are available for pregnant women and children. A detailed study is encouraged for such populations [117].

### 6.5. WHO Indications for Molnupiravir

Only individuals with non-severe COVID-19 at the greatest risk of hospitalization should be given molnupiravir [118]. Molnupiravir users should have a strategy for contraception, and healthcare systems should make pregnancy tests and contraceptives available at the point of treatment. As it is at a developmental stage, less safety data are available. Active monitoring for drug safety is recommended by the WHO, along with other strategies to reduce potential harm [119]. Despite not being generally accessible, attempts have been made to improve access, such as signing a voluntary license agreement. A constrained number of COVID-19 tools are made accessible to nations with access issues via the access to COVID-19 Tools Accelerator [120]. A number of molnupiravir manufacturers are now undergoing evaluation, and prequalification is demanded from them by the WHO. Countries will have a wider variety of products and more affordable costs as a result of the increased number of WHO quality-assured producers.

## 7. Conclusions and Future Perspectives

The current COVID-19 pandemic poses astounding risks to public health, which have been aggravated by the onset of upcoming SARS-CoV-2 variants [121]. Omicron is stated to have 70 times higher possibilities and notable replication when compared to wild type or delta, which may be the reason for the excessive transmission of the disease through oral and nasal routes [122]. Even in the occurrence of higher mutation and replication rates, studies in the UK and South Africa have stated that the need for hospitalization is minor, corresponding to omicron infection. One study also suggested that omicron is derived by the modification of the parent molecule pangolins and hence is not associated with the race of evolution in SARS-CoV-2 [123]. Molnupiravir is a promising orally available anti-SARS-CoV-2 regimen with acceptable safety and an acceptable profile. It inhibits the viral RdRp enzyme, preventing viral chain elongation and ultimately virus survival. It is also considered a broad-spectrum antiviral agent [124,125]. As per the available data from clinical studies, an 800 mg dose twice daily for 5 days is considered effective and safe for COVID-19 patients. Molnupiravir was found to be effective in adult and aged populations infected by delta and omicron variants of SARS-CoV-2. It has also been found to be tolerated in vaccinated individuals and kidney-transplanted recipients. However, it was also observed during a preliminary study that molnupiravir increased the risk of fetal toxicity and developmental deformities. It has not been evaluated thoroughly for infants/children, pregnant women, and the majority of comorbid conditions. Therefore, further work could be done to conduct maximum clinical trials to postulate the mentioned hypothesis and to conclude molnupiravir as a definitive treatment option for COVID-19.

## Figures and Tables

**Table 1:** Comparison of antiviral therapeutics for COVID-19.

	Drug	Molnupiravir	Paxlovid (Nirmatrelvir/Ritonavir)	Remdesivir	Favipiravir	References
Criteria						
Developed by		Merck & Ridgeback	Pfizer	Gilead Sciences	Toyama Chemical Co., Ltd. (Tokyo, Japan)	
Chemical class		Nucleotide analog, prodrug	Peptidomimetic, active drug	Nucleotide analog, prodrug	Nucleotide analog, prodrug	[42]
Mechanism		RdRp inhibitor	Protease inhibitor	RdRp inhibitor	RdRp inhibitor	[43]
Indication		Mild to moderate to severe nonhospitalized high-risk COVID-19 patients	Mild to moderate nonhospitalized adult and pediatric COVID-19 patients	Mild to moderate to severe hospitalized high-risk COVID-19 patients	Mild to moderate hospitalized COVID-19 patients	[43]
Approval status		EUA by US-FDA	EUA by US-FDA	Approved by US-FDA	Approved by Russian Health Ministry and National Medical Products Administration of China, not approved by US-FDA	[44]
Dose		800 mg, twice a day for 5 days	300 mg Nirmatrelvir + 100 mg Ritonavir, twice a day for 5 days	100 mg, once a day for 5 days	1600 mg, twice a day for first day, followed by 600 mg, twice a day for second to fifth days	[45]
Administration route in the body		Oral	Oral	Parenteral	Oral	[46]
Interaction potential		Not the inhibitors or inducers of major drug metabolizing enzymes	Nirmatrelvir is a substrate for CYP3A and P-gp. Ritonavir is a potent CYP3A inhibitor, and is given with nirmatrelvir to increase plasma levels and half-life of nirmatrelvir.	It is an inducer of CYP1A2 and potentially CYP3A4 *in vitro*.	Weak inhibitor of CYPs 1A2, 2C9, 2D6, 2EA, and 3A4; Showed little of no induction of CYPs 1A2, 2C9, 2D6, 2EA, and 3A4	[47]
Cardiac effects		The effect on QT interval yet to be accessed	No effect was observed on QT interval	A possible risk of QT prolongation	The risk of QT elongation is considered low.	[48]
Adverse effects		Diarrhea, nausea, and dizziness	Dysgeusia, diarrhea, hypertension, myalgia, anaphylaxis, and other hypersensitivity reactions	Nausea, hypersensitivity, increase prothrombin time	Diarrhea, liver toxicity, hyperuricemia	[49]
Drug–drug interaction		Not found	Significant drug–drug interaction	No drug–drug interaction studies conducted clinically	Interaction observed with some drugs like chlorpromazine and quetiapine	[47]
Important clinical trial		MOVe-OUT	EPIC-HR	PINETREE	NCT04434248, NCT04529499	[50]
Major concern		Safety concern	Multiple drug–drug interactions	Expensive and parenteral route of administration	High dose required	[51]

**Table 2:** Impact of molnupiravir on SARS-CoV-2 variants of concern.

Variants	Sub Variants	Major Mutations in Spike Protein	Impact of Molnupiravir	References
Delta	B.1.617.2	T19R, G142D, FR156⁃157del, R158G, L452R, T478K, D614G, P681R, D950N	Studies shows that molnupiravir and its metabolite (EIDD-1931) inhibited the VeroE6-GFP cells in the SARS-CoV assay.	[76]
	B.1.617.2		The *in vivo* study for the efficacy of molnupiravir in ferrets and dwarf hamster model indicate that it reduced the lung viral load and prevented transmission.	[78]
	B.1.617.2		The early treatment of mild to moderately infected SARS-CoV-2 unvaccinated adult patient with molnupiravir decreases hospitalization and probability of death by 7.3%.	[79]
	B.1.617.2.1 OR AY.1	K417N, A1146T, V70F, and W258L	Although molnupiravir is recommended by USFDA, specific study related to B.1.617.2.1 is required to prove its efficacy and safety.	[90]
Omicron	B. A1	P681H, A67V, H655Y, S371L, and N679K	The susceptibility of molnupiravir was found to be 0.43 ± 0.08(IC_50_). This proves its effectiveness against B. A1.	[91,92]
	B. A2	S371F, D405N	A lower mortality rate, lower risk of SARS-CoV-2 progression, and redundancy in oxygen therapy was reported in molnupiravir-treated groups compared to the matched controls.	[85]
	B. A2	S371F, D405N	The overall viral RNA clearance was decrease in molnupiravir group.	[86]
	B. A2	P132H, S371F, D405N	The therapeutic results indicate that molnupiravir and its metabolite (EIDD-1931) inhibit the cytopathogenicity in VeroE6-GFP cells.	[93]
	B. A3	A67V, H69del, V70del, T95I, V143del, Y144del, Y145del, N211I, L212del, S371F, D405N and G446S	Although molnupiravir is recommended by USFDA, specific study related to BA.3 is required to prove its efficacy and safety.	[94]
	B. A4	Del69-70, 44 L452R, F486V, and R493Q	Use of molnupiravir is recommended by Japan government along with two other drugs, but clinical trials are not performed to support the data.	[95,96]
	B. A5	Del69-70, 44 L452R, F486V, and R493Q	Use of molnupiravir is recommended by Japan government along with two other drugs, but clinical trials are not performed to support the data.	[95,96]
Hybrid Variant	XD	E172D	A detailed study is required to prove the efficacy of molnupiravir against XD hybrid variant.	[97]
	XE		A detailed study is required to prove the efficacy of molnupiravir against XD hybrid variant.	[97]
	XF		A detailed study is required to prove the efficacy of molnupiravir against XD hybrid variant.	[97]
